# The ERβ4 variant induces transformation of the normal breast mammary epithelial cell line MCF-10A; the ERβ variants ERβ2 and ERβ5 increase aggressiveness of TNBC by regulation of hypoxic signaling

**DOI:** 10.18632/oncotarget.24134

**Published:** 2018-01-10

**Authors:** Michelle Faria, Samaneh Karami, Sergio Granados-Principal, Prasenjit Dey, Akanksha Verma, Dong S. Choi, Olivier Elemento, Tasneem Bawa-Khalfe, Jenny C. Chang, Anders M. Strom, Jan-Åke Gustafsson

**Affiliations:** ^1^ University of Houston, Department of Biology and Biochemistry, Center for Nuclear Receptors and Cell Signaling, Science & Engineering Research Center, Houston, Texas, USA; ^2^ Department of Medical Oncology, Hospital of Jaen, Jaen, Spain; ^3^ Department of Cancer Biology, The University of Texas MD Anderson Cancer Center, Houston, Texas, USA; ^4^ Department of Genomic Medicine, The University of Texas MD Anderson Cancer Center, Houston, Texas, USA; ^5^ Institute for Computational Biomedicine, Department of Physiology and Biophysics Weill Cornell Medicine, New York, NY, USA; ^6^ Methodist Cancer Center, Houston Methodist Hospital, Houston, Texas, USA; ^7^ Department of BioSciences and Nutrition, Karolinska Institutet, Huddinge, Sweden

**Keywords:** slug, twist1, SOX2, CD133, c-Myc

## Abstract

Triple negative breast cancer (TNBC) still remains a challenge to treat in the clinic due to a lack of good targets for treatment. Although TNBC lacks expression of ERα, the expression of ERβ and its variants are detected quite frequently in this cancer type and can represent an avenue for treatment. We show that two of the variants of ERβ, namely ERβ2 and ERβ5, control aggressiveness of TNBC by regulating hypoxic signaling through stabilization of HIF-1α. RNA-seq of patient derived xenografts (PDX) from TNBC shows expression of ERβ2, ERβ4 and ERβ5 variants in more than half of the samples. Furthermore, expression of ERβ4 in the immortalized, normal mammary epithelial cell line MCF-10A that is resistant to tumorsphere formation caused transformation and development of tumorspheres. By contrast, ERβ1, ERβ2 or ERβ5 were unable to support tumorsphere formation. We have previously shown that all variants except ERβ1 stabilize HIF-1α but only ERβ4 appears to have the ability to transform normal mammary epithelial cells, pointing towards a unique property of ERβ4. We propose that ERβ variants may be good diagnostic tools and also serve as novel targets for treatment of breast cancer.

## INTRODUCTION

Triple negative breast cancer continues to be the most difficult form of breast cancer to treat with few options besides chemotherapy [1–3]. Hypoxic signaling appears to play a crucial role during cancer progression initiating a more stem cell like character of the cancer cells leading to chemotherapy and radiation resistance [4–14]. It has been shown that presence of HIF-1α is a negative prognostic factor for breast cancer both in lymph node positive and negative tumors [5, 9, 15–20]. Estrogen receptor β (ERβ) was discovered and cloned in 1996 [21], and shortly after, in 1998, a splice variant of ERβ, ERβ2, was cloned that has a truncated ligand-binding domain (LBD) and a spliced-in unique exon [22]. This variant, which is primate- specific, cannot bind to estrogen or to a classical estrogen response element (ERE). Subsequently, other variants were cloned like ERβ4 and ERβ5, all with a truncated LBD, spliced-in unique exon and unable to bind estrogen. Currently, no known function in normal physiology has been described for these variants, which appear to be expressed only in cancer and not in normal tissue, indicating that they have a function during development but not in adult life. These variants have for a long time been considered as non-functional or inhibitory factors to ERβ1. We have recently shown that they bind to and stabilize both HIF-1α and HIF-2α in prostate cancer cells, thus activating hypoxic signaling under normoxic conditions and furthermore are recruited to HIF-1α response elements in chromatin [23]. Interestingly, ERβ1 has been shown to decrease HIF-1α signaling by up-regulating expression of prolyl dehydrogenase 2 (PDH2) in prostate [24]. Furthermore, ERβ1 has been shown to inhibit NF-kB signaling by causing down regulation of IKKβ through less activation by HIF-1α [25]. NF-kB correlates with worse prognosis of breast cancer in many studies [26]. NF-kB and HIF signaling are also involved in chemotherapy resistance, where one important drug resistance gene is ABCG2/BCRP, which is a target of both HIF and NF-kB signaling [27, 28].

PDX are often used as a model for clinical cancer although passaging can make the PDX differ from the original cancer in composition of cells. However, RNA-sequencing of early passage PDX can give expression profiles of genes associated with cancer progression and lead to discovery of factors important for breast cancer progression. Tumorsphere assay is a widely used functional assay that enriches for cells with enhanced tumor initiation ability [29, 30], where the normal mammary epithelial cell line MCF-10A is often used as a tool to evaluate the transforming ability of a factor by performing tumorsphere assay [31]. In this report we find that many genes regulated by the ERβ variants are direct targets of either HIF-1α or HIF-2α indicating a potentiation of hypoxic signaling, which has previously been shown to affect breast cancer aggressiveness [4–7, 10, 12, 13, 16, 17, 20]. In addition, by analyzing PDX subjected to RNA-seq we found that ERβ4 and ERβ5 are the most widely expressed variants. Expressing ERβ4 in normal mammary epithelial cells stimulates anchorage independent growth indicating a transformation ability.

## RESULTS

### Expression of ERβ variants in PDX from breast cancer and in triple negative breast cancer cell lines

**Figure 1:**
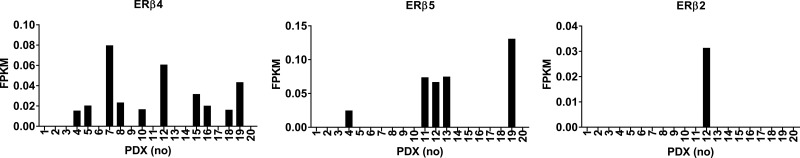
Analysis of RNA-seq data from breast cancer PDX for expression of ERβ2, ERβ4 and ERβ5 The data is presented as Fragments per Kilobase of Exon per Million Fragments Mapped (FPKM).

**Figure 2:**
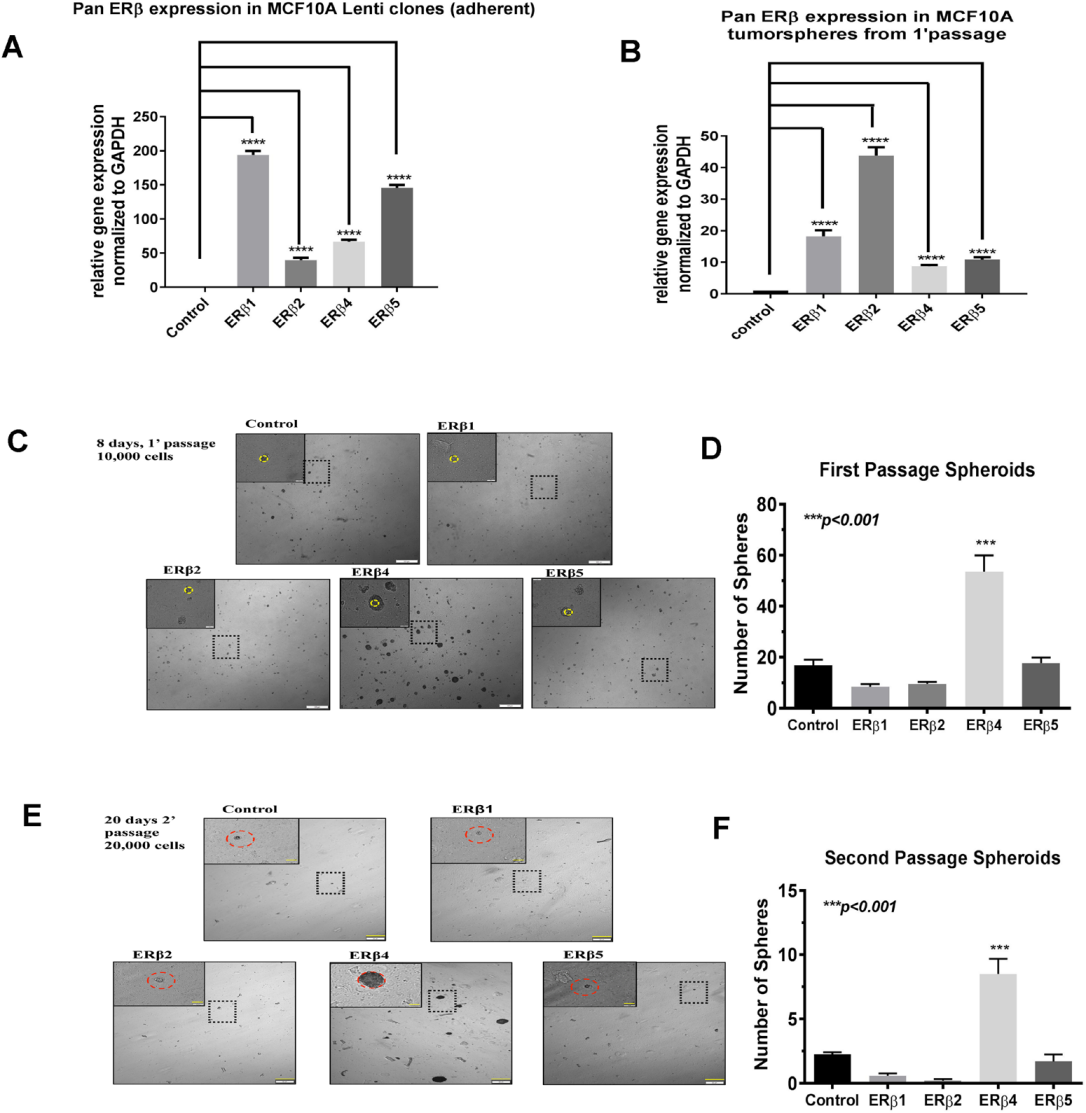
(**A**) Real time PCR was used to evaluate expression of ERβ variants in adherent MCF-10A cells before grown as spheres in the mammosphere assay. (**B**) Real time PCR was used to evaluate expression of ERβ variants in the 1’ generation of mammospheres after 8 days. (**C**) Mammosphere formation of 10,000 single MCF-10A cells was evaluated after 8 days in spheroid suspension. Each image represents the population of stable MCF-10A clones; the yellow circle highlights the average size of spheroids observed in Control-clones compared to spheroids in the respective ER-β variant clones. (**D**) For 2 independent experiments, the number of spheroids in first passage studies was evaluated in 5 random images for each MCF-10A clone. Statistical significance was evaluated with ANOVA and Tukey’s posthoc test. (**E**) First passage spheroids were reconstituted in a single-cell suspension and 20,000 cells were grown in spheroid conditions to establish second passage mammospheres. The red circle illustrates the average size of spheroids observed in ER-β4 MCF-10A clones. (**F**) Second passage spheroids (clusters > 5 cells) were counted in 5 random images and subject to statistical analysis via ANOVA and Tukey’s posthoc test.

We analyzed expression of ERβ variants ERβ2, ERβ4 and ERβ5 in 20 breast cancer PDX with RNA-seq. We excluded ERβ3 which has not been found in cell lines or tumor samples by us or others [32]. We found the variants to be expressed in 12 out of 20 samples, with ERβ4 expressed in 10 samples and ERβ5, in 5 samples, and ERβ4 and ERβ5 co-expressed in 3 samples. ERβ2 was expressed in only one PDX, which also expressed ERβ4 and ERβ5 (Figure 1). Patient primary tumor and xenograft characteristics as well as subtype did not show any correlations to expression of ERβ4 or ERβ5 (See Supplementary Table 1). Only one PDX # 12 is Her2+ and expresses ERβ2, ERβ4 and ERβ5, all other PDX is of basal type. In order to investigate if the ERβ variants could transform normal mammary epithelial cells, we used lentivirus transduction to overexpress the ERβ variants in MCF-10A cells and performed tumorsphere assay. Figure 2A shows expression of variants in the adherent cell line and Figure 2B shows expression in the 1’ generation of suspended cells and tumorspheres. Since neither control, ERβ1, ERβ2 nor ERβ5 expressing MCF-10A cells form tumorspheres the graph shows expression of ERβ1, ERβ2 or ERβ5 in small aggregates of cells not defined as tumorspheres, while expression of ERβ4 occurs in tumorspheres. The MCF-10A cells do not naturally form spheroids since they have a very small population of stem cells [33]. However, we observed that ERβ4 expressing MCF-10A cells formed spheroids (Figure 2C–2D, for higher magnification pictures see Supplementary Figure 1). This observation indicates that the induction of ERβ4 is sufficient to support anchorage-independent growth or transformation of non-cancerous mammary epithelial cells. To evaluate whether ERβ variants also impact self-renewal properties, a single cell suspension of first passage spheroids were regrown in mammosphere conditions to generate second generation spheroids. Only ERβ4 expression could propagate spheroid formation (Figure 2E–2F).

Furthermore, we analyzed endogenous expression of ERβ variants in 4 different TNBC cell lines, BT-549, MDA-MB-231, SUM159 and HCC1806 using quantitative PCR. The variants ERβ1, ERβ2, ERβ4 and ERβ5 were expressed at different levels in all cell lines except SUM159 cells, where expression of all variants was very low. The only cell line expressing all four variants endogenously was HCC1806. We then compared the expression of the variants in SUM159 cells to the rest of the three TNBC cell lines. BT-549 showed expression of ERβ4 and ERβ5, while one of the most studied TNBC cell lines, MDA-MB-231, showed expression of ERβ2 and a low level of ERβ4 (Supplementary Figure 2).

### The variants ERβ2 and ERβ5 regulate genes that correlate to increased aggressiveness of TNBC

**Figure 3:**
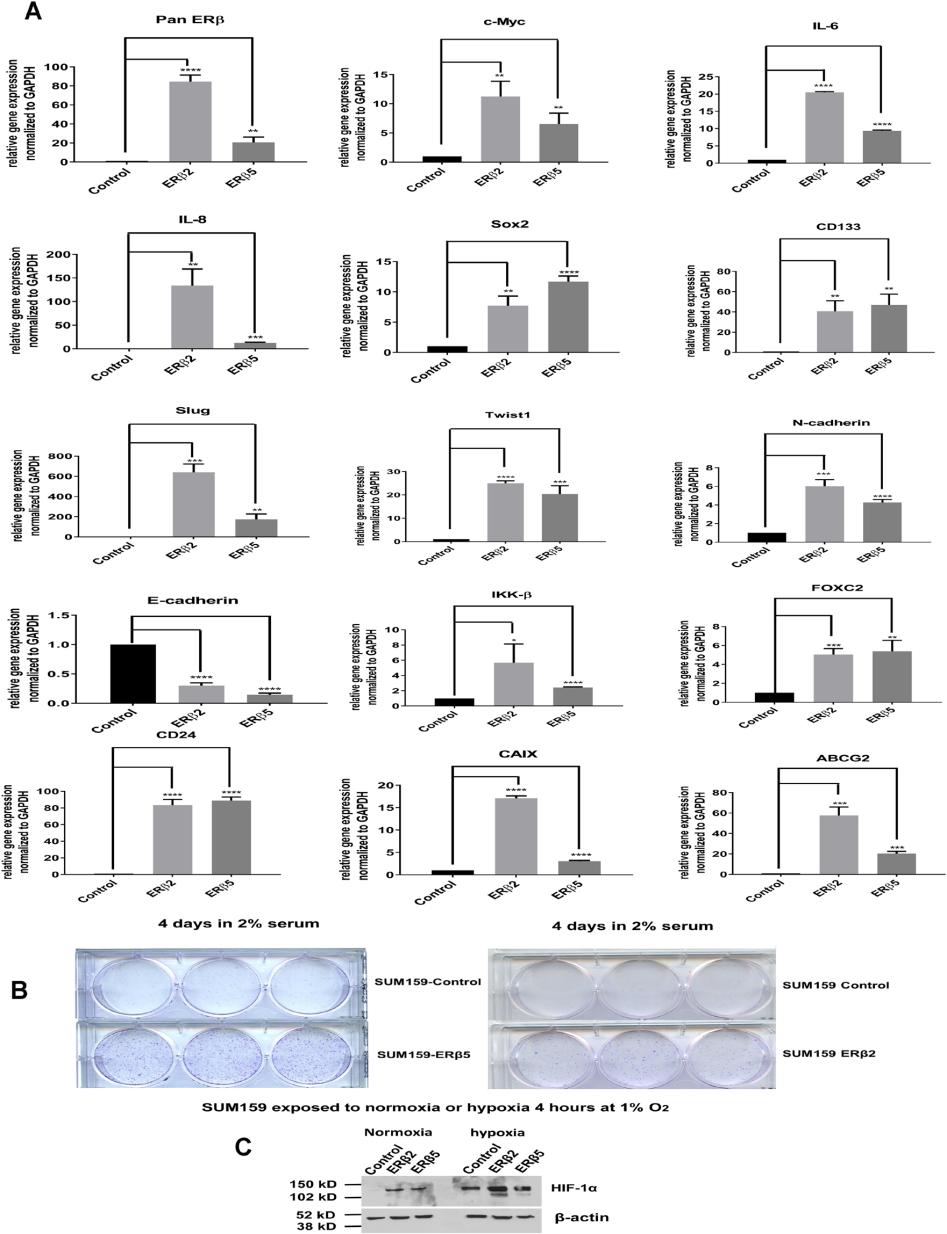
(**A**) SUM159 cells transfected and selected for stable expression of ERβ2 and ERβ5. Real time PCR was used to analyze changes in signaling pathways (basal cell factors, proliferative factors, EMT inducing and stem cell associated factors as well as NF-kB signaling). (**B**) 500 cells of each SUM159 ERβ2 and ERβ5 were plated on each well in a 6 well plate in media containing 2% serum and incubated for 4 days; the cells were visualized with crystal violet staining. (**C**) Western blot of Control, ERβ2 and ERβ5 expressing SUM159 cells exposed to normoxia or hypoxia for 4 hours at 1% O_2,_ detection by HIF-1α antibody.

We used the SUM159 cell line, which expresses low levels of ERβ variants, since both ERβ2 and ERβ5 have been shown to correlate to clinical outcome of breast cancer [34–36], we chose to stably express the ERβ2 and ERβ5 variants in order to study changes in gene regulation. As expected from our previous study in prostate [37], we found many HIF-1α and HIF-2α targets being strongly regulated by ERβ2 and ERβ5 [37] (Figure 3A). Many of the genes regulated have previously been shown to correlate with aggressiveness of breast cancer such as CD24, SOX2, Slug, Twist1, CD-133, N-Cadherin, E-Cadherin, FOXC2, ABCG2, c-Myc, IL8, IL6, and IKKβ (activating NF-κB signaling). It is interesting to note that many of these genes regulate epithelial mesenchymal transition EMT and stem cells. In a previous study, the growth of the SUM159 cells was shown to be dependent on IL8 and IL6 expression maintained by high serum concentration [38]. Therefore, we investigated if expression of ERβ2 and ERβ5 in the SUM159 cells at low serum concentration could maintain the cell growth under these conditions. We found that ERβ2 and ERβ5 can maintain the cell growth at low serum using a growth assay (Figure 3B). In addition, we found that expression of both ERβ2 and ERβ5 increases HIF-1α protein expression under normoxic condition and potentiates hypoxia induction of HIF-1α, establishing a connection between the variants and hypoxic signaling (Figure 3C).

### Expression of ERβ variants changes proliferation of TNBC cell lines

**Figure 4:**
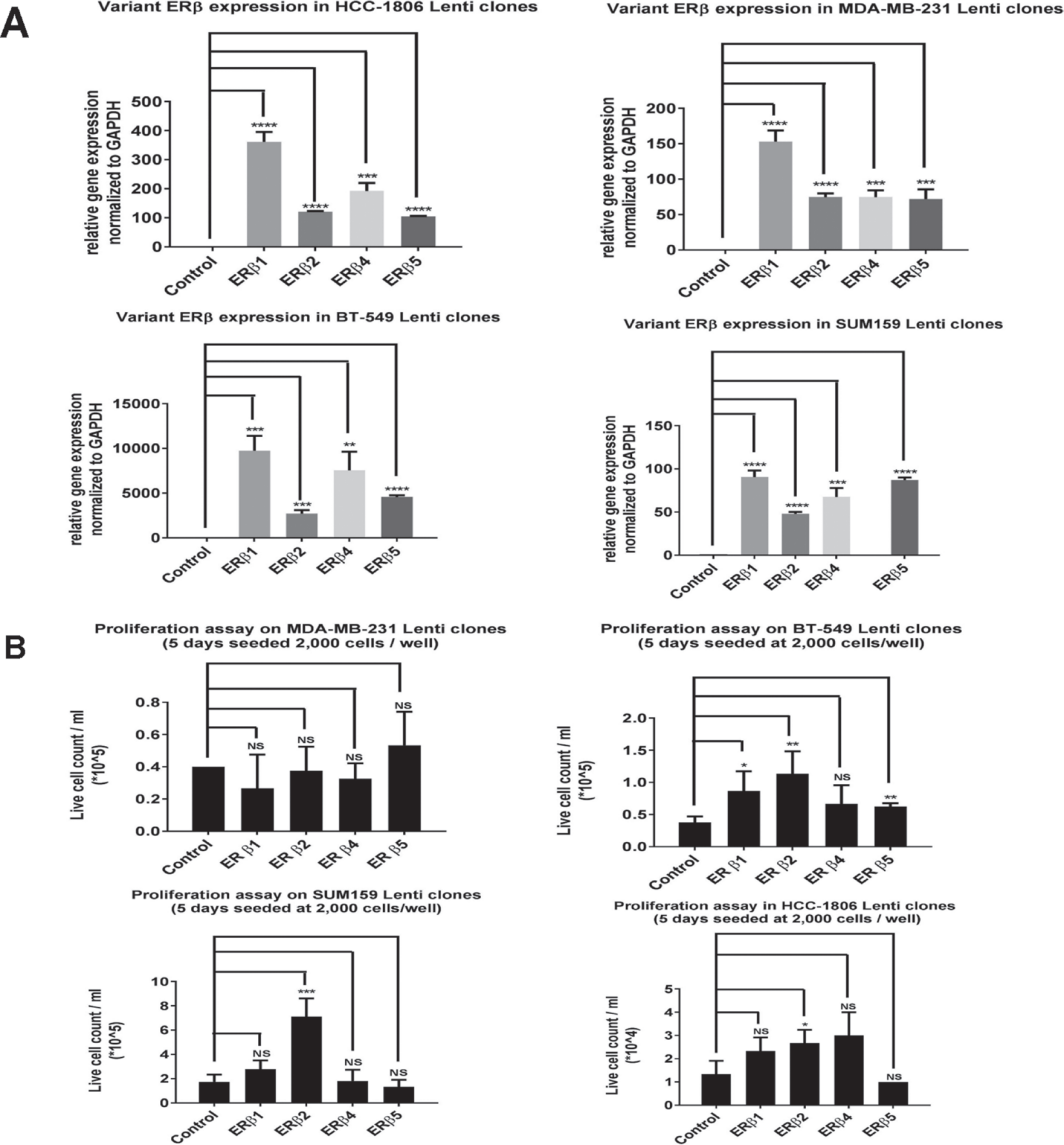
(**A**) Real time PCR was used to evaluate ERβ variant expression level in the different TNBC cell lines transduced with lentivirus. (**B**) For proliferation assays 2,000 cells were plated onto triplicates of wells in a 6 well plate into the normal media supplemented with 10% serum. After 5 days at 37°C, 5% CO_2_, the cells were trypsinized and counted using countess.

To investigate the effect on proliferation by ERβ1, ERβ2, ERβ4 and ERβ5, we used lentivirus transduced SUM159, MDA-MB-231, BT-549 and HCC1806 cells to express the variants at higher levels and compared to cells transduced with empty lentivirus (Figure 4A). TNBC cell lines showed various effects on proliferation with the different variants, however, ERβ2 was most proliferative across all cell lines (Figure 4B). As can be seen in Supplementary Figure 3, there is a pronounced effect on colony formation by ERβ2, and less effect by ERβ4 and ERβ5 in the SUM159 cells. No change in colony formation compared to control was observed for ERβ1 in SUM159 cells. In the MDA-MB-231 and BT-549 cells, expression of all variants showed no change from control.

**Figure 5:**
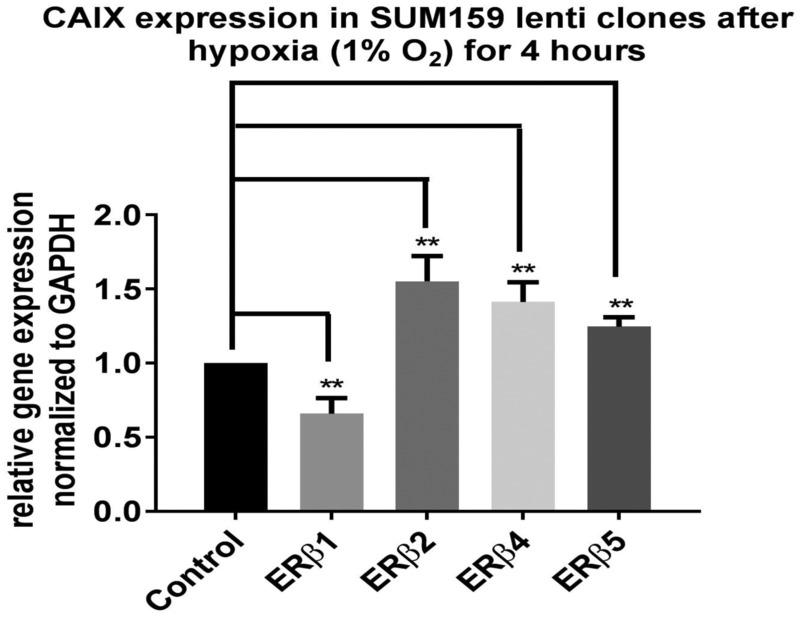
SUM159 cells expressing different ERβ variants were exposed to hypoxia 1% O_2_ for 4 hours RNA was prepared and analyzed using real time PCR for analysis of regulation of the hypoxia-induced CA-IX.

Exposing the SUM159 cell lines expressing the different ERβ variants to hypoxia shows that ERβ1 reduces hypoxic induction of carbonic anhydrase (CAIX) which is a HIF-1α regulated gene; this is in agreement with ERβ1’s regulation of prolyl dehydrogenase 2 increasing degradation of HIF-1α. On the other hand, ERβ2, 4 and 5 increase hypoxia-induced levels of CAIX, which is in agreement with our previous studies showing stabilization of HIF-1α by ERβ2 [37] (Figure 5).

## DISCUSSION

Although TNBC lacks expression of ERα, PR and HER2-neu receptors, our data indicates that ERβ variants are expressed in at least 50% of the TNBC cell lines at mRNA levels. These variants except ERβ1 are primate-specific with no known ligands, In addition, the variants ERβ2, ERβ4, and ERβ5 appear to be cancer specific i.e. they are not expressed in normal tissue in the adult, which makes them very suitable targets for treatment because a specific drug can potentially show less side effects by having less effect on normal tissue.

Our studies indicate that ERβ variants can increase aggressiveness of TNBC by promoting hypoxic signaling under normoxia. It is interesting to note that nuclear ERβ2 correlates to worse prognosis in ERα negative breast cancer [39]. Another study of more than 2000 breast cancer samples showed that expression of ERβ5 was a marker of worse outcome in Her2 positive and TNBC [36]. In a previous study in prostate cancer, we have shown that ERβ2 is recruited to HIF response elements in Twist1 and VEGF genes using ChIP-assay [37]. Thus, it appears that ERβ variants take advantage of hypoxic signaling by stabilizing HIF-1α and HIF-2α protein expression to maintain a conducive environment for carcinogenesis. Hypoxic signaling is an adaptive stress response, which activates downstream targets involved with angiogenesis, glucose metabolism, etc. for promoting tumor growth in a low-oxygen, necrotic environment. A recent report by Samanta *et al.* showed that hypoxia inducible factors are required for chemotherapy resistance of breast cancer stem cells [8]. Expression of HIF-2α can regulate stem cell populations, which would promote tumor cell self-renewal and differentiation into suitable cell lineages to benefit tumor growth [40]. As a strong indication of up-regulated HIF signaling, we observed induction of carbonic anhydrase (CAIX), which is a gene that is dependent on HIF-1α for its expression [41]; in addition, expression of CAIX in breast tumors correlates to poor prognosis [42]. We found up-regulation of SOX2 by both ERβ2 and ERβ5. SOX2 expression has been found to be positively associated with TNBC and metastatic breast cancers. Higher SOX2 expression level was found to be correlated with poorer outcomes in TNBC patients [43, 44]. In addition, we found up-regulation of Slug, which is an upstream regulator of SOX2 [44]; expression of Slug is associated with basal-like breast cancer [45]. We also found increased expression of c-Myc in cells expressing ERβ2 or ERβ5; increased c-Myc expression correlates to bad prognosis in breast cancer [46]. It is interesting to note that HIF-1α and HIF-2α have been shown to have opposing effects on transcription of the c-Myc promoter, an effect that has been attributed to the observation that HIF-1α binds to the C-terminal domain of β-catenin thus interfering with recruitment of the co-activator p300, while HIF-2α binds to the N-terminus of β-catenin, thus increasing recruitment of p300 and allowing transcription to occur [47]. Over-expression of twist is associated with markers of EMT and predicts poor prognosis in breast cancers via ERK and Akt activation and facilitates bone metastasis [48, 49]. Another regulated factor, CD133, is associated with vasculogenic mimicry (VM) in TNBC, and is correlated with lymph node positivity and high-grade tumor. The close relationship between CD133 expression and VM might be a key for tumor relapse and progression [50]. The cell surface factor CD24 has been shown to be an effector of HIF-1α driven primary tumor growth and metastasis [51]. We also observe the classical indication of EMT by decreased E-Cadherin and increased N-Cadherin, a switch which is associated with tumor progression and metastasis. In addition, we found that IL-8 and IL-6 were increased by both ERβ2 and ERβ5. It is interesting to note that IL-8 has been shown to increase the cancer stem cell population in pancreatic cancer and increase tumorsphere -forming phenotype [52]; IL-8 has also been shown to increase the cancer stem cell population in breast cancer [53–55]. We also found upregulation of FOXC2 by ERβ2 and ERβ5; expression of FOXC2 is associated with claudin-low/basal B breast tumors or other EMT-/CSC-enriched tumors [56]. Tumors often have hypoxic regions expressing HIF-1α. We found that the variants affected HIF-1α expression during normoxia and under hypoxia by a strong potentiation of HIF-1α expression when ERβ2 and ERβ5 were expressed in the SUM159 cells. This indicates that even a mild hypoxia where the variants are expressed could give a survival advantage to the cells. It is interesting to note that a recent paper by Huang et al. [57] shows that ERβ2 expression was associated with hypoxic regions in clinical breast cancer samples. In agreement with this we have observed that ERβ2 is also stabilized by hypoxia or by HIF-1α expression (data not shown).

**Figure 6:**
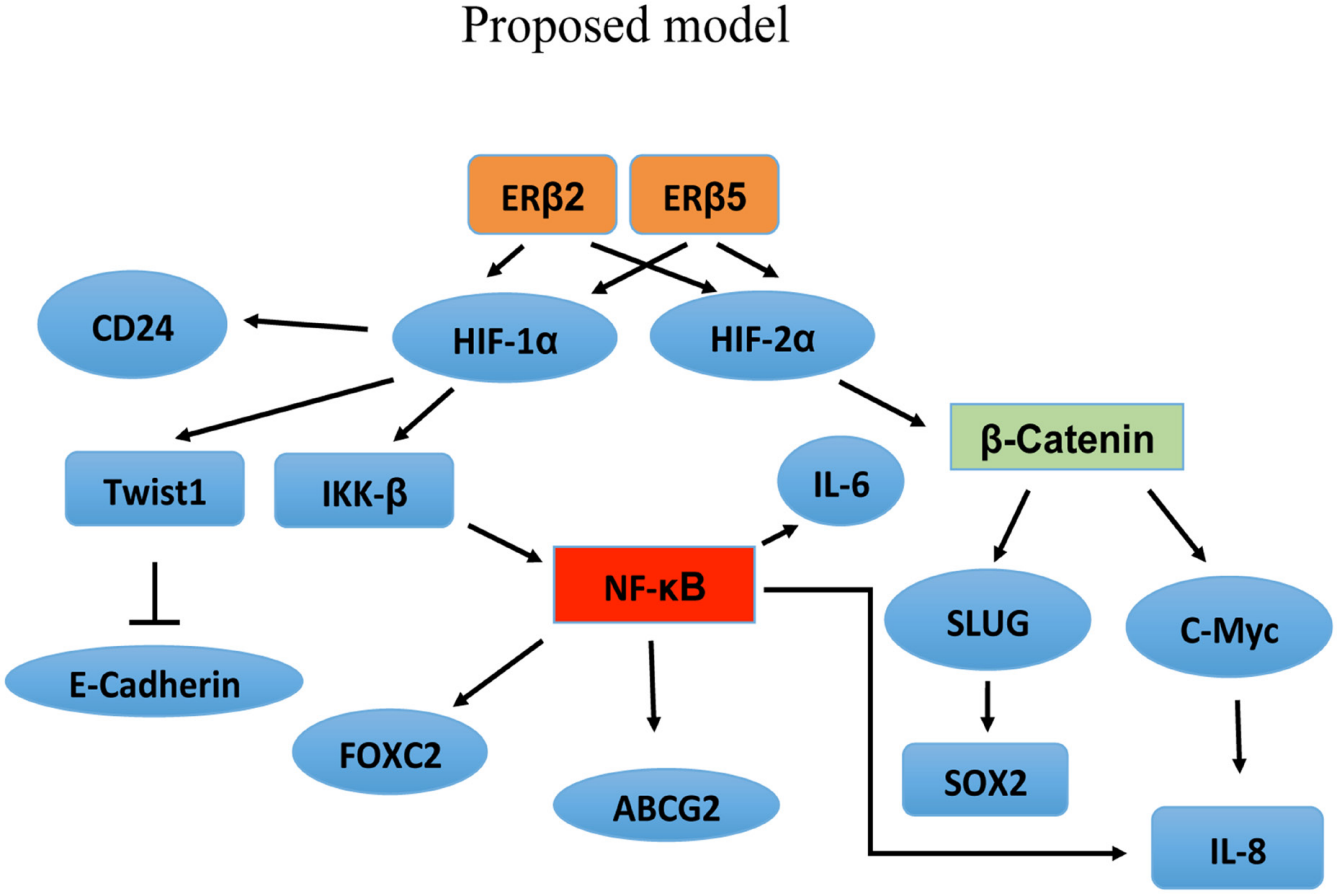
Proposed model showing that the observed gene regulation can be explained by the ability of the ERβ variants to interact with and stabilize HIF-1α and HIF-2α

The up-regulation of ABCG2, an important drug efflux transporter gene (53), by ERβ2 and ERβ5, indicates that ERβ variants could contribute towards chemo-resistance. Since ERβ1 has been shown to decrease hypoxic signaling and the variants increase this signaling there could be a delicate balance between the ratios of ERβ1 and ERβ variants at different stages of TNBC progression to allow EMT and migration of tumor cells to form metastases and achieve chemotherapy resistance. We find expression of two of the variants, ERβ4 and ERβ5, in more than 50% of PDX from breast cancer (*n* = 20). It is also interesting to note that ERβ4 induces tumorsphere formation in the immortalized mammary epithelial cell line, MCF-10A. The MCF-10A cells are not forming tumorspheres indicating that the ERβ4 variant has a transforming ability and can increase the stem cell population. We find that ERβ4 and ERβ5 are expressed at a low level in the PDX, however this could be explained by the fact that only a small number of cells are expressing the factors at low levels which could be cancer stem cells. It is interesting to note that although ERβ4 is transforming MCF-10A cells, it does not appear to increase proliferation of TNBC cell lines, while ERβ2 which is not transforming the MCF-10A cells, is more potent in stimulating proliferation of TNBC cell lines. One can speculate that ERβ4 is involved at an early stage of tumor development to expand cancer stem cells, but not needed later to drive tumor progression. Another interesting observation is that ERβ2 seems to stimulate proliferation in cell lines showing low endogenous expression of ERβ2, like BT-549 and SUM159 cells. A hypothetical overview is presented in Figure 6, linking the variant stabilization of HIF-1α and HIF-2α to the effect on gene regulation. More studies are needed to evaluate the full importance of ERβ variants in TNBC and outlining the mechanism of mammary epithelial transformation by ERβ4 possibly leading to use of the variants as drug targets or prognostic factors.

## MATERIALS AND METHODS

### Cell culture and generation of stable ERβ-expressing cells

The HCC1806, BT-549 and MDA-MB-231 cell lines were obtained from the American Type Culture Collection (ATCC) and maintained in RPMI-1640 (Invitrogen Inc., Carlsbad, CA) medium supplemented with 10% fetal bovine serum (FBS) (Sigma, St. Louis, MO), 2 mM L-glutamine, and 25 mM HEPES buffer (Invitrogen Carlsbad, CA). The MCF-10A cell line was given by Dr. Christoforos Thomas. The SUM159 cell line was a gift from Dr. Cecilia Williams and was maintained in DMEM (Invitrogen Inc., Carlsbad, CA) medium supplemented with 10% fetal bovine serum (FBS) (Sigma, St. Louis, MO). For ligand treatment, the medium was changed to phenol red-free RPMI-1640 supplemented with 2% dextran-coated charcoal-treated FBS (DCC) (Sigma, St. Louis, MO). All experiments used the cells below passage 30.

### PDX model

The PDX model was developed as described by Zhan et al. [58]

### RNA-Seq alignment and quantification

Raw sequenced reads were aligned to the Human reference genome (Version hg19 from UCSC) using STAR (Version 2.4.2) aligner. Aligned reads were quantified against the reference annotation (hg19 from UCSC) to obtain FPKM (Fragments per Kilobase per million) using CuffLinks (v 2.2.1).

### Construction of an inducible system for ERβ2 and ERβ5

We used the breast cancer cell line SUM159, that expresses low levels of endogenous ERβ2 or ERβ5 to investigate gene regulation of the ERbeta variants. To introduce expression, we used a transposon-based system, stably transfected into SUM159 breast cancer cells with the ERβ constructs (SUM159-ERβ2 and SUM159-ERβ5) or with empty vector (SUM159-control) as a control. The transposon-based system consisted of two plasmids: (a) pIR-TRE, which contains a pair of transposon elements (IR) to allow the efficient integration of the plasmid and the gene (ERβ2 or ERβ5) into the genomic DNA, and (b) piggybac, which expresses the transposase enzyme required for the integration of the transposon element. The pIR-TRE plasmid also contained a bi-directional promoter expressing the GFP gene in one direction and the tetracycline-regulated ERβ (Tet-off) gene in the other direction. The transactivator for Tet-off system regulation via the tetracycline response element (TRE) was from a third plasmid, pTAN. The ERβ genes were cloned into the PvuII/NheI (Promega, Madison, WI) site of pIR-TRE. All of the plasmids were transfected using Lipofectamine 2000 (Invitrogen Inc., Carlsbad, CA) in Opti-Mem medium (Invitrogen Carlsbad, CA) according to the manufacturer’s protocol. Complete DMEM medium with 10% FBS medium was added after 6 h of incubation with the transfection reagents.

### Lentivirus transduction of cells producing stable cells

As an alternative expression system we cloned all the FLAG tagged ERβ variants ERβ1, ERβ2, ERβ4 and ERβ5 into lenti6-TOPO and infected the 5 different cell lines SUM159, HCC1806, MDA-MB-231, BT-549 and MCF-10A with virus at 2 m.o.i. The cells were selected with 5 µg/ml of blasticidine for at least one week before analysis.

### Colony formation assay

Cells were seeded onto a 6 well plate at a density of 500 cells/well in media containing 10% FBS; for HCC1806, RPMI1640 was used and for SUM159, DMEM was used. The plates were incubated at 37°C in 5% CO_2_ atmosphere for one week and then stained with 0.5% crystal violet, 6% glutaraldehyde in water.

### Tumorsphere assay of MCF-10A

Tumorsphere assay was performed as described in Karami et al., “Novel SUMO-Protease SENP7S Regulates β-catenin Signaling and Mammary Epithelial Cell Transformation” Scientific Reports, April 24, 2017.

### RNA extraction and qPCR

RNA extraction was performed with RNeasy (Qiagen, Valencia CA) according to the manufacturer’s protocol. cDNA was synthesized from 1 μg of the total RNA with the iScript First-Strand System according to the standard protocol (BioRad, Hercules, CA). Real-time PCR was performed with the iTaq master mix (BioRad, Hercules, CA). The primers used are given in Supplementary Table 1. All the primers were ordered from Integrated DNA Technologies, Inc. (Coralville, IA). The qPCR reactions were performed with a 7500 Fast Real-Time PCR System (Applied Biosystems Foster City, CA) using optimized conditions for the iTaq dye system: 50°C for 2 min, 95°C for 10 min, followed by 40–50 cycles at 95°C for 15 sec and 60°C for 50 sec. The optimum concentration of primers was determined in preliminary experiments, and the amplification specificity was confirmed by dissociation curve analysis.

### Statistics

The values are expressed as the mean with 95% confidence intervals. An unpaired two-tailed *t*-test was used to compare the differences between two groups. The significance is presented as ^*^P < 0.05, ^**^P < 0.005, and ^***^P < 0.001, ^***^P < 0.0001 and non-significant differences are presented as NS.

## SUPPLEMENTARY MATERIALS FIGURES AND TABLE



## Abbreviations

TNBC: triple negative breast cancer
PDX: patient derived xenograft
LBD: ligand binding domain of receptor
