# Supplementary material for: The ERβ4 variant induces transformation of the normal breast mammary epithelial cell line MCF-10A; the ERβ variants ERβ2 and ERβ5 increase aggressiveness of TNBC by regulation of hypoxic signaling
Source: Oncotarget. 2018 Jan 10;9(15):12201–11. doi: 10.18632/oncotarget.24134 (PMC5844739; doi:10.18632/oncotarget.24134)
Supplement: Supplementary file 1 [file oncotarget-09-12201-s001.pdf]

The ERβ4 variant induces transformation of the normal breast mammary epithelial cell line MCF-10A; the ERβ variants ERβ2 and ERβ5 increase aggressiveness of TNBC by regulation of hypoxic signaling

SUPPLEMENTARY MATERIALS

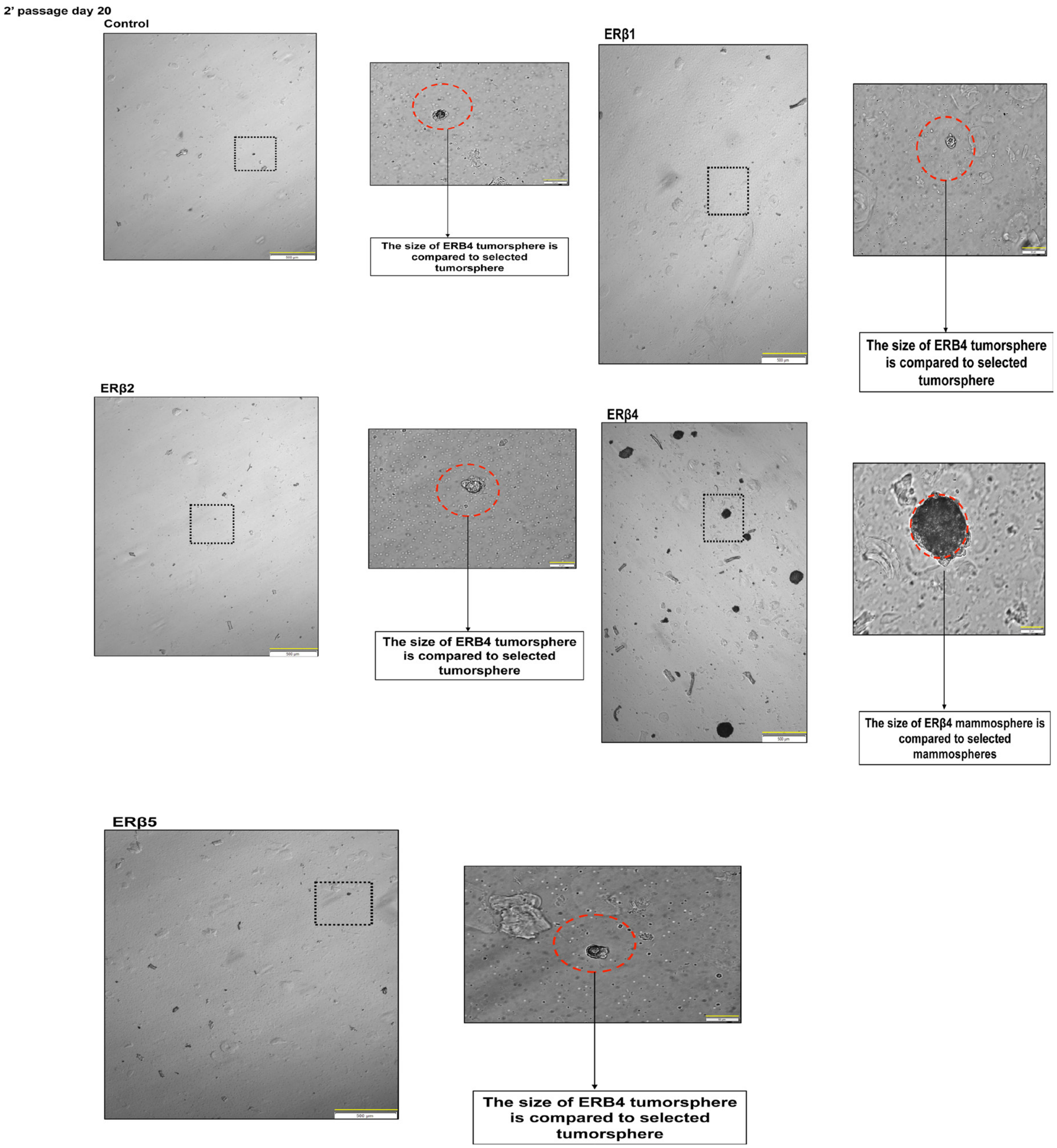

Supplementary Figure 1: Higher magnification of spheroids after 6 days and 20 days 1' generation of spheroids.

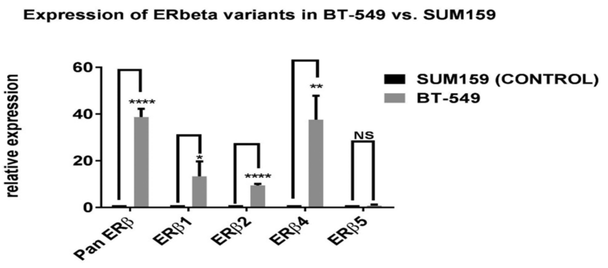

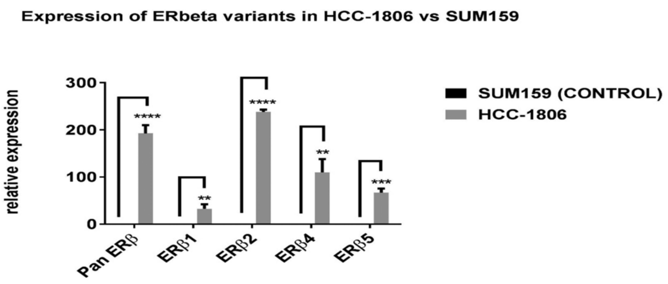

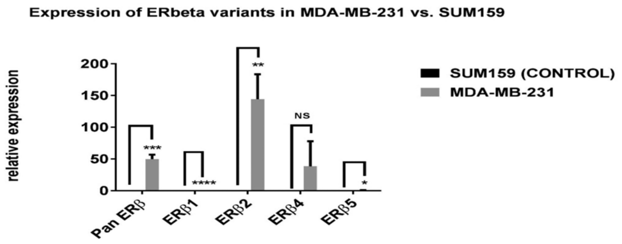

Ct values

Cell line	PanERβ	ERβ1	ERβ2	ERβ4	ERβ5
SUM159	32.0	34.0	33.0	33.0	30.0
HCC1806	27.7	32.0	28.4	29.6	28.1
BT-549	29.8	34.0	32.8	30.9	34.0
MDA-MB-231	28.0	33.1	27.6	30.0	33.5

Supplementary Figure 2: Expression of mRNA for ERβ1 and variants ERβ2, ERβ4 and ERβ5 in 3 different TNBC cell lines BT-549, MDA-MB-231 and HCC1806 compared to expression in SUM159.

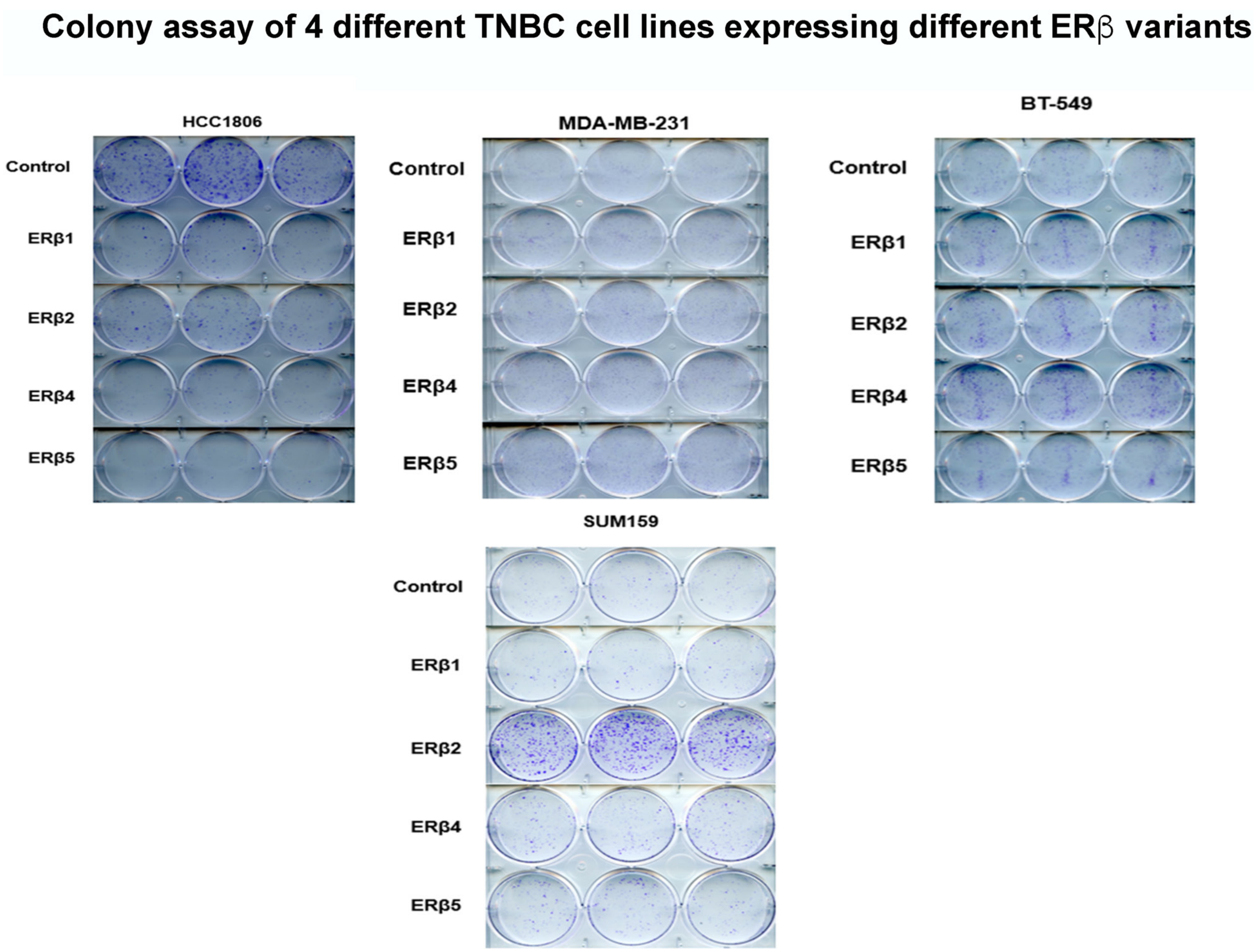

Supplementary Figure 3: For colony formation assay, 500 cells were plated onto triplicates of wells in a 6 well plate into the normal media supplemented with 10% serum. After 7 days at 37°C, 5% CO_2 the cells were fixed and stained in 0.5% crystal violet solution containing 6% glutaraldehyde for 20 minutes at room temperature.

Supplementary Table 1: Clinical data describing treatment and response

Sample#	PDX ID	Subtype	ERβ2	ERβ4	ERβ5	Treatments in patients	Clinical Response	Xenograft Metastasis Rate To Mouse Lung
1	BCM2147	TNBC/basal				AC	Res	0
2	BCM2665	TNBC/basal				AC, Doc	AC Sen, Doc. Res	7.1
3	BCM3107	TNBC/baal				Doc	Sen	0
4	BCM3204	TNBC/basal		+	+	AC	Res	28.6
5	BCM3561A	TNBC/basal		+		nd	nd	0
6	BCM3611	TNBC/basal				AC + GSI –> Doc + GSI	AC + GSI Res; Doc + GSI Res	0
7	BCM3807	TNBC/basal		+		AC	Res	0
8	BCM3887	TNBC/basal		+		Xeloda (5FU)	Res	14.3
9	BCM3904	TNBC/basal				Doc	Res	18.2
10	BCM3936	TNBC/basal		+		AC	Sen	9.1
11	BCM4013	TNBC/basal			+	Das + Doc	Res	21.4
12	BCM4175	HER2+	+	+	+	Das –> AC	Res	0
13	BCM4195	TNBC/basal			+	Das –> Doc	Das Res; Doc Res	0
14	BCM4272	TNBC/basal				GSI + Doc	Sen	28.6
15	BCM4664	TNBC/basal		+		Das + Doc	Das Res; Doc Res	0
16	BCM4698	TNBC/basal		+		nd	nd	nd
17	BCM4913	TNBC/basal				Doc	Res	0
18	HMCC5310B	TNBC/basal		+		nd	nd	nd
19	BCM5471	TNBC/basal		+	+	GSI + Doc	Sen	33.3
20	BCM5998	TNBC/basal				AC	Res	0

Abbreviations, AC: doxorubicin (Adriamycin) and cyclophosphamide (Cytoxan); Doc, Docetaxel; GSI, gamma secretase inhibitor; Das, Dasatinib; Xeloda (Capecitabine, 5-Fu), Res: resistant. Sen: sensitive, nd: not determined.
